# Legal Notes for Institutional Workers

**Published:** 1911-11-25

**Authors:** 


### LEGAL NOTES FOR INSTITUTIONAL WORKERS.

**(The Editor will be pleased to answer Legal queries in this column.)**

### Liabilities of Hospital Governors.

It is somewhat disheartening to those who have devoted time and trouble to institutional management to find themselves made defendants to an action for negligence. Just as the disappointed litigant sometimes blames his lawyer, so the patient who has not been cured visits the failure upon those who did what they could to assist him, and if there is a choice in the matter, he issues his writ against those who have the longest purse. Fortunately, however, the judges of the land have intervened to protect the governors of institutions from this particularly vexatious kind of action. An interesting case, which shows that protection is afforded, even in cases where an action is brought by a paying patient, was decided in the Court of Session on November 5. The facts are very brief. The pursuer fractured her thigh bone on February 19, 1910. On the following day she entered the Greenock Infirmary as a "paying patient"; two guineas a week was charged for board and lodging. She was treated for a sprain of the knee-joint followed by synovitis. After leaving the institution on March 21, 1910, she brought an action against its "office bearers and directors" for £1,000 damages for negligence. She stated that if the doctors employed had acted with reasonable skill, they would have discovered the fractured thigh. The action was dismissed by Lord Skerrington, and the Division affirmed his decision. Lord Dundas pointed out that the medical staff were not the servants of the governing body, and that, if the plaintiff was to succeed she must rest her claim on a contract express or implied. In the absence of express contract, however, the authorities only undertook to furnish the services of a competent staff; they did not undertake to indemnify patients against errors of treatment. He added: "On paying two guineas a week she was to receive board on the scale usually supplied to paying patients, and medical and surgical treatment available to patients in the infirmary—that was, the service of a competent staff of medical men."

The reasoning which decided this case is wholly satisfactory to hospital authorities. It has been held by the English Court of Appeal that the governors of a great public hospital like St. Bartholomew's are not liable to non-paying patients for the negligence of a "competent staff." The same principle has now been applied—in Scotland—to a paying patient. As the greater includes the less, it may be assumed that there is a clear consensus of judicial opinion as to the liability of hospital governors on both the south and north sides of the Tweed.

